# Three synchronous, sporadic and separate periampullary and pancreatic tumors: more than a coincidence?

**DOI:** 10.1186/1477-7819-12-382

**Published:** 2014-12-13

**Authors:** Amit Sastry, Michael Wayne, Justin Steele, Mazen Iskandar, Songyang Yuan, Avram M Cooperman

**Affiliations:** Department of Surgery, Mount Sinai Beth Israel, 37 Union Square West, 4th floor, New York, NY 10003 USA; Department of Pathology, Mount Sinai Beth Israel, 1st ave at 16th St, New York, NY 10003 USA

**Keywords:** Pancreas, Synchronous, Pancreatic adenocarcinoma, Neuroendocrine tumor, Adenosquamous

## Abstract

Three sporadic, synchronous, and separate lesions in the ampulla of Vater and the head of the pancreas presented in an 81-year-old male. One was symptomatic and two were incidental. One was detected preoperatively (the ampullary lesion) and two by examination of the resected specimen (the neuroendocrine and pancreatic carcinomas). The case is summarized and the literature and the issue of commonality are reviewed.

## Background

Multiple primary neoplasms (MPNs) develop in 5 to 10% of the population [1–4]. Synchronous lesions are identified within 2 to 6 months of the primary tumor and metachronous lesions after 6 months [5]. MPNs may be in the same organ (infrequent) or different organs (much more frequent).

Three synchronous, sporadic, separate tumors involving the head of the pancreas are very rare (<0.5%) [5, 6], and suggest a causal relation. In our case, two of the three were malignant - an adenosquamous cancer of the ampulla of Vater, and an adenocarcinoma of the head of the pancreas - while the third, a neuroendocrine tumor (NET), had “benign” features. We summarize the case, and review the literature and possible connections between lesions.

## Case presentation

An 81-year-old male presented with painless jaundice of 1 week duration, preceded by nausea, anorexia and 4.5 kg weight loss. There was a history of coronary artery disease and type II diabetes mellitus. Abnormal laboratory tests included a bilirubin of 11.1 mg/ dl, an alkaline phosphatase of 481 U/L, and an aspartate and alanine transaminase ratio of 303/262 U/L. Magnetic resonance imaging showed a 1.6-cm cystic neoplasm in the head of the pancreas. An endoscopy, endoscopic retrograde cholangiopancreatogram, and endoscopic sonogram revealed a bulbous ampulla suggesting a submucosal mass, which was confluent with a mass in the head of the pancreas. It was 2.6 cm, solid and irregular, and obstructed a dilated bile and pancreatic duct. The impression was a pancreatic or ampullary malignancy. The surgical impression was a pancreatic malignancy invading the ampulla and duodenum and, if health risks permitted, surgery was advised. A biliary stent was placed at endoscopy, the jaundice cleared, and after medical and cardiac clearance the patient underwent surgery. Absent of metastases, a single lesion was palpated in the head of the pancreas near the ampulla. A pylorus preserving pancreaticoduodenectomy was performed in January 2013. The patient’s postoperative course was uneventful. He was discharged on postoperative day 9. Despite chemotherapy, hepatic metastases were noted by June 2013 and had increased by August 2013. He died in November 2013, 10 months after surgery.

### Pathology

**Figure 1:**
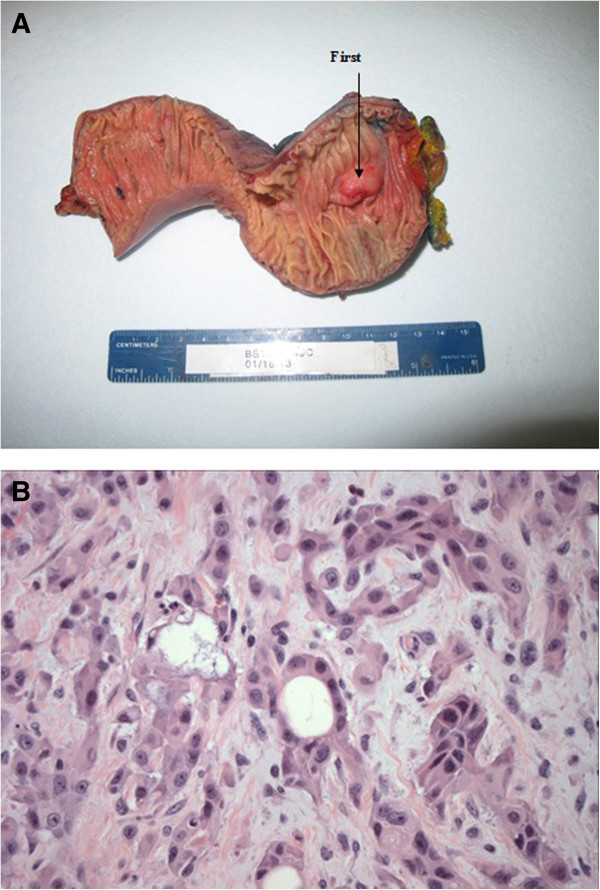
**Adenosquamous carcinoma of the ampulla of Vater. (A)** Gross image of tumor. **(B)** Solid nests with focal glandular formation. Hematoxylin and eosin stain, 400×.

**Figure 2:**
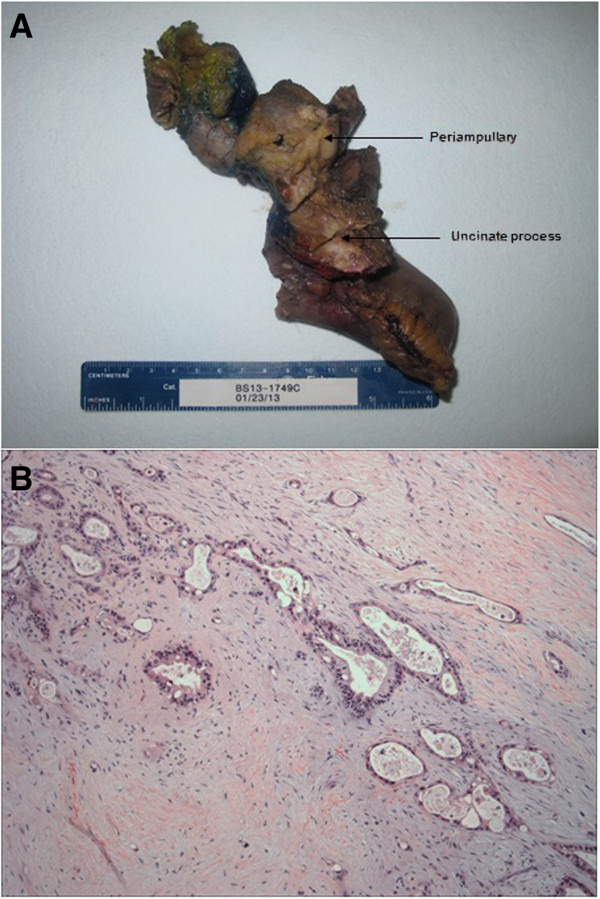
**Adenocarcinoma of uncinate process. (A)** Gross image of tumor with periampullary tumor above it. **(B)** Tumor consists of variable sized and shaped invasive tumor glands in the background diffuse stoma fibrosis. Hematoxylin and eosin stain, 100×.

**Figure 3:**
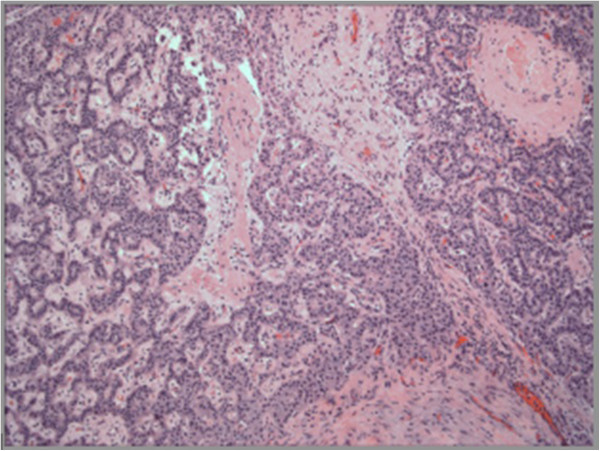
**Neuroendocrine tumor of the anterior pancreas.** Tumor shows the characteristic trabecular pattern and monotonous cells of endocrine neoplasm. Hematoxylin and eosin stain, 100×.

The gross and histological images are shown in Figures 1, 2 and 3. The periampullary tumor (Figure 1) was a 3.5-cm, poorly differentiated adenosquamous carcinoma with lymphovascular and perineural invasion with metastases in one out of 7 lymph nodes (T3N1). The tumor cells expressed p63 and ck5/6 but not chromogranin and synaptophysin.

In the uncinate process, a second lesion (Figure 2) was identified 1 cm from the ampullary lesion. It was a pancreatic ductal adenocarcinoma, 3.1 cm in size and moderately differentiated, with perineural and peripancreatic fat invasion. There was no lymph node or lymphovascular invasion (T3N0).

The third lesion in the anterior pancreatic head (Figure 3) was a 1.2-cm, well differentiated NET with cystic degeneration, but no lymphovascular or perineural invasion (T1N0). The mitotic rate was <2 mitoses/50 HPF (High-power-field), and the tumor cells were positive for chromogranin and synaptophysin with a low proliferation rate by Ki67 of 1%.

An additional finding throughout the resected specimen was chronic pancreatitis and high grade pancreatic intraductal neoplasia (PanIN 3).

**Figure 4:**
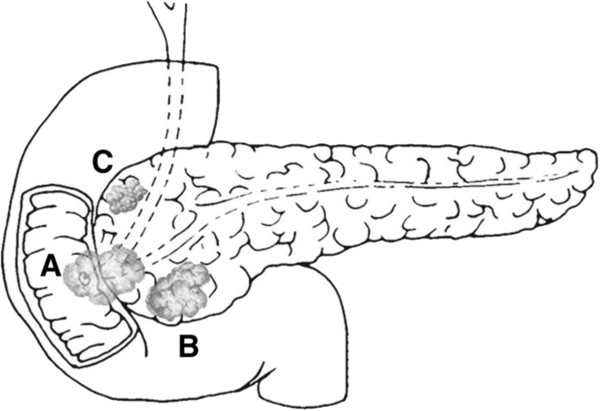
**The relationship of the three tumors in the head of the pancreas. A**, the adenosquamous ampullary cancer; **B**, the adenocarcinoma of the head of the pancreas; **C**, the neuroendocrine tumor.

A diagram depicting the location and relation of each tumor in the head of the pancreas is provided in Figure 4.

## Discussion

MPN are not common, with an incidence of 5 to 10% [1–4, 6]. The prevalence of MPNs is increasing because of several factors, including longer lifespans, widespread use of body imaging, longer survival of many cancer patients, lifestyle choices (smoking and alcohol), genetics, environment, nutrition and immunology [7]. MPNs are synchronous or metachronous. The definitions are arbitrary, implying detection within 2 or 6 months (synchronous) or after 6 months (metachronous) from the primary tumor [8]. A 44-month mean interval from treatment of the primary tumor until detection of metachronous tumors in one registry suggests that the longer the survival after the primary tumor, the greater the chance for metachronous lesions [9]. MPNs infrequently develop in the same organ. When MPNs develop after exposure to the same risk factors in different organs, they are designated as “field cancerization” (for example, head and neck, and breast-ovary and urogenital cancers [2, 10]). Patients who develop MPN in unrelated organs are “cancer prone”. Dedifferentiation of cells from exocrine to ductal epithelial cells, and the loss of alleles 3p and 10q on chromosome 10 are present in 40 to 50% of pancreatic serous cystadenomas and some pancreatic adenocarcinomas [11]. This may be a shared pathway for the development of either lesion [12–15]. Chronic inflammation with high-grade PanIN lesions which were pervasive in the resected specimen are premalignant and are associated with a higher risk for pancreatic adenocarcinoma [16]. Some “unrelated” tumors are clonally related [17]. Unique to our patient are three separate, synchronous pancreatic epithelial lesions simultaneously detected. This is very rare (prevalence of <0.5%) [3]. A third of synchronous MPNs detected simultaneously occur in related organs (breast-breast, breast-ovary, ovary-endometrium, pancreas-biliary tree) [18, 19].

Multiple reported synchronous pancreatic lesions include combinations of adenocarcinoma, NETs, and cystic lesions. Usually two lesions are identified. Synchronous pancreatic adenocarcinomas and intraductal papillary mucinous tumors (IPMNs) have been reported (most are side-branch lesions) to coexist in 9% of cases [20]. One publication reported multi-centric pancreatic adenocarcinomas and a side branch IPMN. The patient initially presented with an incidental side-branch IPMN, unchanged for 6 years. A pancreatic duct irregularity (a 1.2-cm cancer) was noted and the patient underwent a distal pancreatic resection for a small carcinoma. Intraoperative cytology after lavage of the remaining pancreatic duct revealed malignant cells. The patient underwent total pancreatectomy and three other adenocarcinomas were found: two were *in situ* and the third was a 3-mm invasive lesion. Despite the small size of the invasive lesions, and normal markers, one metastatic node was present [21]. Many MPNs share exposure to the same risk or genetic factors [2, 10]. In the pancreas, centroacinar and duct cells differ in cells of origin but allele losses on chromosome 10q are present in 50% of serous cyst adenomas as well as some adenocarcinomas [11]. The pathway involves mutations of the phosphatase and tensin homolog gene, a tumor suppressor gene [15]. Adenocarcinoma of the pancreas may also coexist with gallbladder and/or bile duct cancer [22, 23]. When two synchronous lesions are detected the symptomatic lesion is primary, and the other lesion is incidental. Exocrine and endocrine epithelial cells have similar morphologic and embryologic origins and endocrine and epithelial tumors coexist in 1.3% of cases [12]. Immunostaining confirms that exocrine cells do differentiate into ductal epithelial tumor cells [12–14]. In our case, chronic pancreatitis with high-grade PanIN 3 was found throughout the resected specimen. By itself, chronic pancreatitis and chronic inflammation leads to pancreatic neoplasia [16]. Although multiple etiologies may coexist in patients with MPN, causality has been difficult to prove.

Ampullary cancer accounts for 1% of all gastrointestinal malignancies, 6% of all periampullary cancers, and are one-tenth as frequent as pancreatic cancer [24]. Ampullary adenosquamous cancers are very rare (perhaps 10 cases have been reported), with a dismal prognosis [25]. Treatment is usually operative, either a pancreaticoduodenectomy or ampullectomy. Survival is usually less than 6 months, although two reported patients survived at 19 and 46 months [26]. Our patient lived 10 months after surgery, although metastases were evident at 5 months. The prognosis for ampullary adenosquamous cancer is worse than for adenocarcinoma of the pancreas. Some suggest that, if the diagnosis is made before treatment, chemoradiation be the initial treatment [27].

The second lesion, a 3.1-cm adenocarcinoma of the pancreas was an incidental lesion, not identified by endoscopic sonogram or magnetic resonance imaging. The dismal prognosis of pancreatic adenocarcinoma is well known. Even when incidental to other lesions or detected by serial screening the prognosis and cure rates are poor [28].

The third lesion was a small (1.2 cm) pancreatic NET with cystic degeneration. As body imaging has become common, small incidental NETs are frequently discovered [28]. Although considered low-grade malignancies, they are slow growing, and generally "well behaved". By itself, a 1.2-cm nonfunctional lesion would be observed. Only 79 malignant pancreatic NETs of the pancreas have been reported since 1973, of which more than three-quarters were metastatic with a 5-year survival of 37.5% [29].

## Conclusions

An 81-year-old male who presented with jaundice was found to have an ampullary cancer and an incidental pancreatic adenocarcinoma and a neuroendocrine cystic lesion. After stenting of the bile duct and medical clearance, a pancreaticoduodenal resection was performed. The postoperative course was uneventful. The pathology report noted an obstructive ampullary adenosquamous cancer - a rare, highly malignant and dismal tumor. Two synchronous lesions, a 3-cm adenocarcinoma of the pancreas, and a 1.2-cm NET were identified as separate lesions in the resected specimen. Metastases were evident at 5 months and the patient died at 10 months.

## Consent

Informed consent was obtained from the patient’s next of kin for publication of this case report and any accompanying images.

## Authors’ information

AS and MI are surgical residents at Mount Sinai Beth Israel. MW, JS, and AMC are attending surgical oncologists at Mount Sinai Beth Israel. SY is an attending pathologist at Mount Sinai Beth Israel.

## Abbreviations

IPMN: intraductal papillary mucinous tumor
MPN: multiple primary neoplasm
NET: neuroendocrine tumor
PanIN: pancreatic intraductal neoplasia.
